# Comparative Assessment of Hygroscopic Properties and Thermal Performance of Activated Carbon-Based Physical Adsorbents and Advanced Composite Adsorbents

**DOI:** 10.3390/ma18102280

**Published:** 2025-05-14

**Authors:** Siyu Wei, Zhengpeng Fan, Songyu Zhang, Yutong Xiao, Chunhao Wang, Shanbi Peng, Xueying Zhang

**Affiliations:** 1 Safety, Environment and Technology Supervision Research Institute, PetroChina Southwest Oil and Gas Field Company, Chengdu 610041, China; 2 School of Civil Engineering and Geomatics, Southwest Petroleum University, Chengdu 610500, China; 3 Dongfang Boiler Co., Ltd., Zigong 643002, China; 4 Zero-Carbon Biofuel Research Center, Tianfu Yongxing Laboratory, Chengdu 610213, China; 5 Chongqing Gas Mine, PetroChina Southwest Oil and Gas Field Company, Chongqing 401120, China

**Keywords:** adsorption heat storage, water vapor, physical adsorbent, chemical adsorbent, exothermic properties

## Abstract

The water adsorption property was shown to be the critical process limiting the thermal output in the adsorption heat storage driven by the air humidity process, which was different for the physical adsorbent and the physical/chemical adsorbent. In this study, coconut shell-based activated carbon (CAC), a hierarchically porous material that is both low-cost and mass-producible, was utilized as a physical adsorbent and as a matrix for loading calcium chloride (CAC/Ca). The incorporation of calcium chloride in CAC, with a 24% content, resulted in a 4~102% increase in water uptake capacity. The water uptake dynamics of high-thickness adsorbents are inhibited, especially for CAC/Ca. In the context of the adsorption test conducted within a fixed-bed reactor, an increase in air velocity was observed to facilitate water vapor supply, thereby culminating in higher output temperatures for both CAC and CAC/Ca, indicating a higher hydration conversion. The maximum discharge powers of CAC/Ca increased from 2 kW/m^3^ to 20 kW/m^3^, with the air velocity increasing from 0.5 m/s to 2.5 m/s. The heat-release densities of CAC and CAC/Ca at the air velocity of 2.5 m/s were 156 kJ/kg and 547 kJ/kg, respectively.

## 1. Introduction

The global energy landscape is undergoing significant transformation driven by the need to mitigate climate change [1,2]. This transition is accelerating the adoption of renewables, like solar and wind, offering sustainable alternatives to fossil fuels [3]. However, the intermittent nature of these sources presents challenges in their utilization [4]. Thermal energy storage (TES) systems address this by storing excess energy during peak production and releasing it during low output or high demand, optimizing renewable energy use [5,6]. Among TES technologies, sorption heat storage has gained attention for its reversible desorption/sorption cycle between sorbents and sorbates [7]. Known for high energy density, low heat loss, and efficient low-temperature operation, sorption systems are particularly effective for long-term solar thermal storage and space heating [8].

Porous materials, such as silica gel [9,10], zeolites [11], and Metal–Organic Frameworks (MOFs) [12], and other materials with micro- or mesoporous pores are used as physical adsorbents. Qiu found that the theoretical heat storage density and desorption activation energy of silica gel were 1029.63 kJ/kg and 66.75 kJ/mol, respectively [9]. Aristov et al. concluded that the activation energy and heat transfer properties of the silica gel/H_2_O system are influenced by the grain size of the silica gel [13]. The water exchange amount of zeolites during the adsorption/desorption process reached as much as 0.4 g/g, with heat storage capacity of 107 to 185 kWh/m^3^ [11]. Permyakova et al. found that the adsorption capacity of MIL-160(Al) was 0.36 g/g (30 °C, 1.25 kPa). MIL-101(Cr) had a higher water capacity of 1 g/g [12]. To improve porous materials for adsorption heat storage, their water adsorption capacity, particularly at low pressures, must be enhanced through structural and chemical modifications.

Hygroscopic salts are capable of thermodynamically elevating water load lift and enhancing heat storage density [11]. CaCl_2_ [14], MgSO_4_ [15], MgCl_2_ [16], K_2_CO_3_ [17], and other salt hydrates [18,19] are thoroughly identified as potential candidates for adsorption heat storage. The hydrated salt adsorption process involves deliquescence [20], which also brings problems such as slow sorption/desorption kinetics and a high pressure drop during sorption [11]. Researchers have developed composite adsorbents by embedding hydrated salts into matrix pores, improving mass transfer during reactions while preventing issues like liquid leakage and agglomeration [21]. Current matrices include zeolites [11,22,23], activated carbon [24], graphite [25,26], MOFs [27,28], minerals [29,30,31], and other materials [32,33,34,35].

Activated carbon (AC), known for its low cost and industrial production from waste biomass, is a promising material for adsorption and energy storage [36,37,38]. Bennici achieved a homogeneous dispersion of MgSO_4_ in AC, reporting hydration enthalpies of 859 J/g-dry material at 30% relative humidity and 1324 J/g-dry material at 60% relative humidity [39]. Yang used hollow activated carbon as a support for LiOH, achieving an energy storage density of 1916.4 kJ/kg due to its multimodal porosity [40]. Among various AC sources, coconut shell-activated carbon (CAC) stands out for its high surface area, availability, and excellent cyclability [41]. Hou utilized CAC and corn cob-derived AC as a matrix for impregnating organic latent thermal materials [42]. Similarly, Hekimoğlu developed novel composite PCMs by integrating methyl stearate with CAC [43]. However, CAC has not yet been investigated for its potential in adsorption heat storage applications.

In this study, CAC was employed for the first time in sorption heat storage, serving both as a physical adsorbent (CAC) and as a matrix for loading calcium chloride to create a composite adsorbent (CAC/Ca). The composition, structural properties, and static adsorption performance of both samples were systematically characterized. The exothermic behaviors of the two adsorbents were evaluated in a fixed-bed reactor to explore the enhancement effect of chemisorption. Additionally, the impact of air velocity on the exothermic power and heat release density of CAC and CAC/Ca was investigated. Finally, the heat storage densities of CAC and CAC/Ca were compared to assess their performance.

## 2. Materials and Methods

### 2.1. Sample Preparation

**Figure 1:**
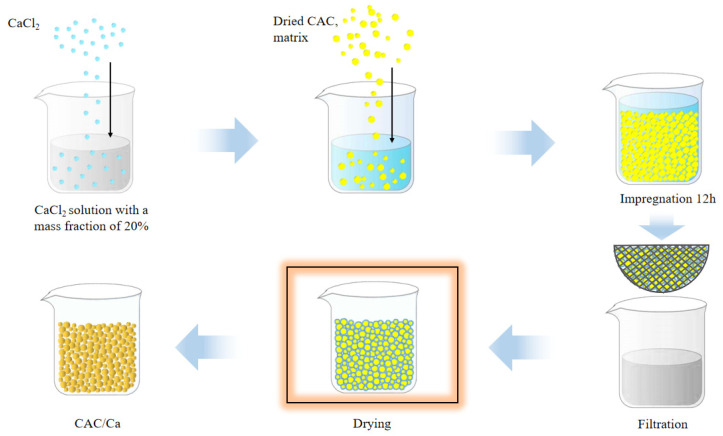
Schematic of the composite heat storage material by the impregnation method.

Calcium chloride anhydrous obtained from Shanghai Aladdin Biochemical Technology Co. (Shanghai, China). Coconut shell-based activated carbon (CAC) was procured from the Sichuan Demo Activated Carbon Company (Chengdu, China). The 2–6 mm particles were then separated by means of sieving. The procedure for preparing the composite sorbent is illustrated in Figure 1. The 20% CaCl_2_ solution was prepared and configured. Meanwhile, the matrix material CAC was subjected to a heating process, initially at 150 °C for four hours and subsequently at 300 °C for two hours, with the objective of removing the physically adsorbed water in the CAC. The dried CAC was infiltrated in the CaCl_2_ solution for 12 h, after which the solid was separated from the solution using a filter. The solid was subsequently dried under ambient air conditions at 80 °C for 2 h to yield the CAC/Ca composite material. Prior to the discharge test, both the CAC and CAC/Ca were subjected to a complete drying process.

### 2.2. Characterization

The porous structure of the samples was examined through the automatic surface characterization analyzer (Micromeritics, ASAP 2460, Norcross, GA, USA). The Brunauer–Emmett–Teller (BET) method was employed to measure the specific surface area of the samples. The compositions of CAC and CAC/Ca were conducted using an X-ray diffraction instrument (PANalytical B.V., X’Pert Pro, Almelo, The Netherlands). Additionally, the morphological features were examined with an emission scanning electron microscope (JEOL Ltd., JSM-7800F, Tokyo, Japan) with an energy-dispersive spectrometer (Oxford, Ultim Max65, Abingdon, UK). The Raman spectrum was recorded on the spectrometer (HORIBA, Xplora ONE, Kyoto, Japan) at 532 nm. The surface functional groups on the samples were examined using a Fourier-transform infrared spectrometer (Nicolet, FTIR-380, Madison, WI, USA). The desorption power and heat absorption enthalpies of the samples were studied using a thermal analyzer (SETARAM, Sensys Evo, Lyon, France). In total, 10 mg of the hydrated sample, obtained from the static adsorption test, with thicknesses of 0.2 cm and a hydration period of 5 h (20 °C, RH 60%), was placed into the measuring apparatus and subsequently subjected to a heating process from 25 °C to 200 °C (5 °C/min, 50 mL/min N_2_).

### 2.3. The Static Adsorption Test

The static adsorption performance was examined in a thermostat–humidistat chamber under 20 °C and 70% relative humidity (RH). The samples were placed in an evaporating dish set up with thicknesses of 0.2 cm, 0.5 cm, and 1.0 cm. Prior to the test, the sample was dried at 150 °C for 4 h. Thereafter, the mass of the sample was meticulously recorded at 30-min intervals. The water uptake w (g/g) was defined as follows: w = (m − m_0_)/m_0_, where m_0_ (g) represents the sample’s mass prior to testing, and m (g) represents the sample’s mass during the experiment.

### 2.4. The Adsorption Test in Fixed-Bed Reactor

**Figure 2:**
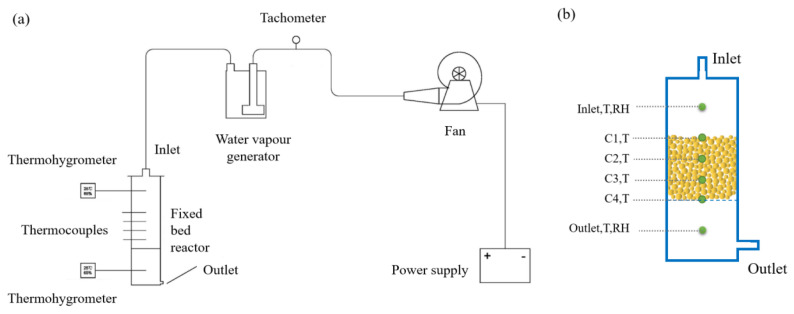
Schematic of the test platform (**a**) and the reactor (**b**).

The heat output test was conducted in a fixed-bed reaction system comprising a fan, tachometer, steam generator, fixed-bed reactor, thermocouples, and thermohygrographs, as illustrated in Figure 2a. The fan was driven by the power supply and produced an airflow at varying velocities. Subsequently, the velocity of the airflow was quantified by a tachometer and conveyed through a hose into a water vapor generator, thereby enabling the generation of a humid airflow. Subsequently, the humid air was introduced into the reactor, where it passed through the bed and exited through the outlet.

As shown in Figure 2b, the reactor features a cylindrical design, measuring 8 cm in internal diameter and 30 cm in height. The reactor is subdivided into three sections, each measuring 10 cm in height. To prevent heat loss, the reactor surface is coated with cotton. The sample was introduced into the reactor’s central region. A thermocouple temperature measurement point, labelled C1, C2, C3, and C4, is positioned at 2.5 cm intervals in order to record the bed temperature. Two thermohygrographs were positioned at the inlet and outlet of the reactor, respectively, to record the temperature and humidity at the inlet and outlet airflows. The different dried samples were introduced into the reactor, which was filled to a total volume of 502.65 cm^3^ for 280 g of CAC and for 334 g of CAC/Ca. Meanwhile, the power supply was activated to generate the air flow (0.5 m/s, 1.5 m/s, and 2.5 m/s), after which the reactor was flooded with humid air. The temperature of the bed, along with the temperature and RH of the inlet and outlet airflows, was recorded. Subsequently, following a 12-h test period, the sample was removed and permitted to dry completely, after which the next sample was introduced.

## 3. Results and Discussion

### 3.1. Material Characterization

**Figure 3:**
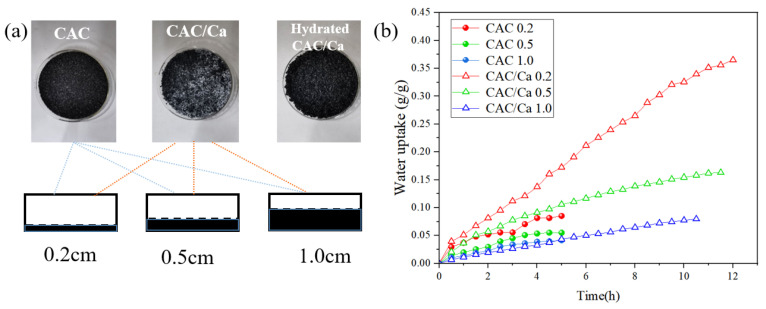
Pictures of CAC, CAC/Ca, and CAC/Ca after adsorption (**a**). Static adsorption curves for samples of different thicknesses (**b**).

Figure 3a shows pictures of CAC, CAC/Ca, and CAC/Ca after adsorption, positioned within evaporating dishes of varying thicknesses. CAC is a black granule with a diameter of 2–6 mm. The surface of CAC/Ca exhibits the precipitation of white calcium chloride, which subsequently reverts to black following the completion of the adsorption process. The static adsorption performance of CAC and CAC/Ca with varying thicknesses is displayed in Figure 3b. The incorporation of calcium chloride in CAC, with a 24% content, resulted in a 4~102% increase in water uptake amount. The water uptake of CAC and CAC/Ca was found to be comparable at a thickness of 1.0 cm, with a value of 0.04 g/g at the fifth hour. However, at a thickness of 0.2 cm, the adsorption rate of CAC/Ca was markedly faster, with a water uptake that was 113% higher than that of CAC at 5 h. In static adsorption, reduced thickness shortens water vapor diffusion paths, enhancing adsorption kinetics for both CAC and CAC/Ca. Notably, CAC/Ca exhibits a salient improvement in adsorption capacity and reaction rates with decreasing thickness—owing to the rapid chemisorption kinetics of calcium chloride, which synergizes with accelerated molecular diffusion. In contrast, pure CAC, relying solely on physisorption-driven van der Waals interactions, shows minimal thickness dependence due to rapid surface saturation at accessible adsorption sites.

**Figure 4:**
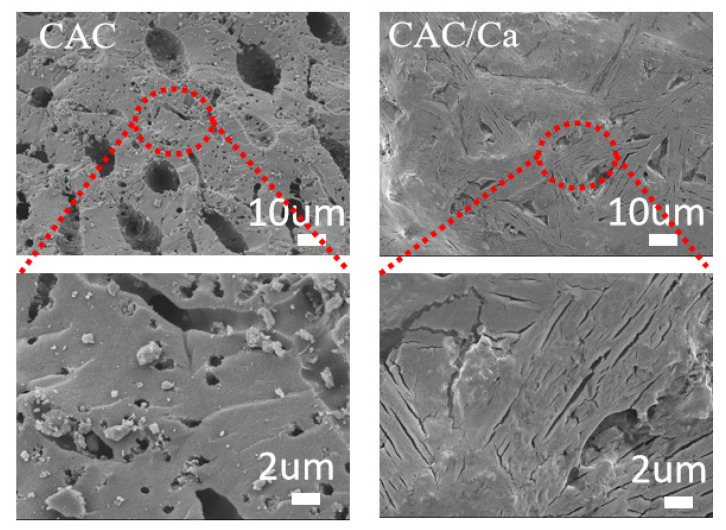
SEM images of the CAC and CAC/Ca.

**Figure 5:**
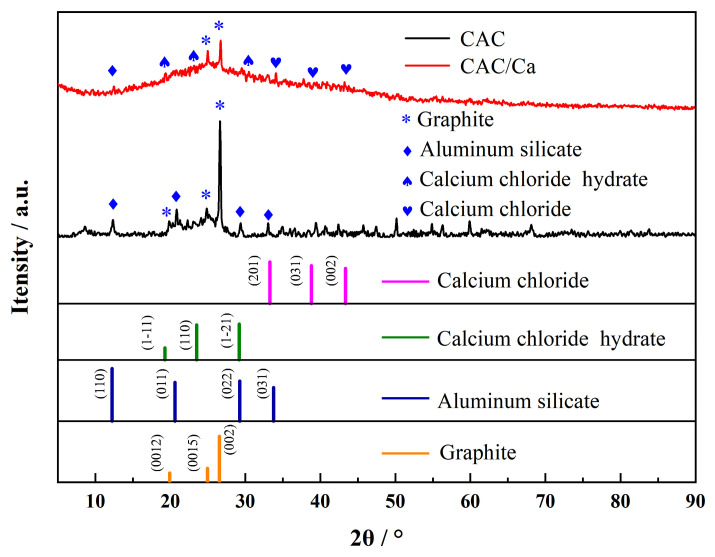
XRD patterns of CAC and CAC/Ca.

Scanning electron microscope (SEM) images of CAC and CAC/Ca are shown in Figure 4. The CAC was observed to comprise micro- and nanoscale holes in the carbon wall, while in the case of CAC/Ca, the matrix was found to be covered by hydrated salt. X-ray diffraction (XRD) patterns and the detailed physical information of samples are shown in Figure 5. The XRD pattern of CAC displays a distinct aluminum silicate peak, accompanied by a multitude of impurity-related peaks. The appearance of peaks corresponding to calcium chloride and calcium chloride hydrate in the CAC/Ca spectrum confirms that the impregnation method successfully formed a composite of calcium chloride and CAC. This evidence indicates the possibility that impurities may have been retained in the commercial activated carbon during the preparation process. The presence of calcium chloride peaks in CAC/Ca indicated that the composite adsorbents had been successfully obtained.

**Figure 6:**
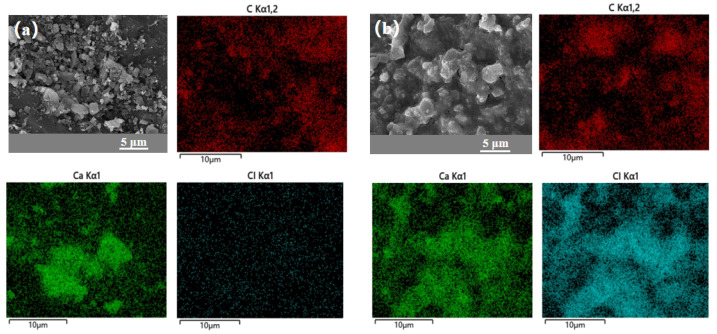
The SEM-EDX images of CAC (**a**) and CAC/Ca (**b**).

The crystalline structure of the CAC is further characterized by Raman spectroscopy analysis (Supplementary Figure S1). The Raman spectra of all the carbons exhibit two broad bands of the D band peak at 1355 cm^−1^ (the disordered carbon or defective graphitic band) and the G band peak at 1590 cm^−1^ (the crystalline graphite band), characteristic of the disordered structure [44]. The FTIR analysis (Supplementary Figure S2) reveals no additional functional groups in the CAC/Ca composite compared to CAC, indicating that the interaction between calcium chloride and CAC is dominated by physical mixture. With the introduction of calcium chloride, the intensity of the IG/ID shows an increasing tendency, which is due to calcium chloride disrupting the sp^2^ network of the carbon skeleton and introducing more defects. The EDX spectrum analysis (Figure 6) reveals that CAC/Ca contains a significant amount of chlorine (Cl) from calcium chloride, whereas CAC shows negligible Cl content. This indicates that the surface of CAC is effectively covered by calcium chloride in the composite material.

**Figure 7:**
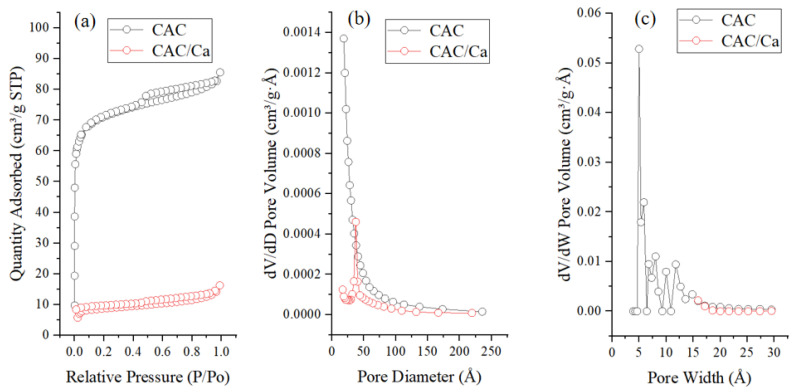
Nitrogen adsorption/desorption isotherm (**a**) and pore diameter (**b**,**c**) of samples.

Figure 7a presents the N_2_ adsorption–desorption isotherms of CAC and CAC/Ca at 77 K. CAC exhibited a Type I isotherm with a distinct hysteresis loop at higher relative pressures (P/P_0_ > 0.4), characteristic of a microporous-dominated material containing limited mesoporosity. In contrast, CAC/Ca showed a collapsed Type I profile with minimal hysteresis, indicating near-complete micropore occlusion and retention of only a small fraction of mesopores. The corresponding pore size distribution curves are shown in Figure 7b,c, with detailed structural parameters summarized in Table 1. CAC had a pore volume of 0.13 cm^3^/g and an average pore size of 3.8 nm, while CAC/Ca exhibited a significantly reduced pore volume (0.03 cm^3^/g) and an increased average pore size (7.0 nm). This structural transformation confirmed that calcium chloride hydrate occupies the micropores of CAC, altering the pore hierarchy while preserving mesopores for efficient vapor transport.

**Figure 8:**
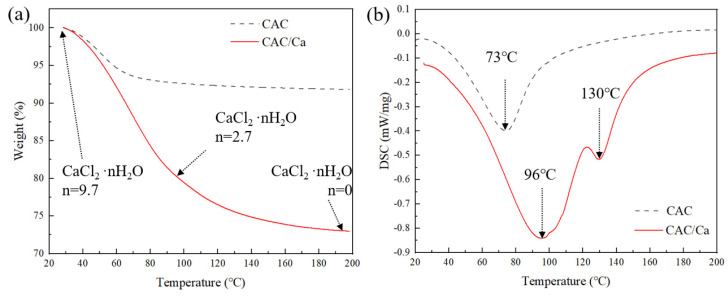
TG (**a**) and DSC (**b**) results of the CAC and CAC/Ca.

The thermogravimetric (TG) curves of CAC and CAC/Ca in Figure 8a show that as the temperature rose, the samples’ weights gradually declined because of water loss. In the case of CAC, a rapid rate of weight loss was observed until the temperature reached approximately 80 °C, after which the rate of weight loss slowed down. At 200 °C, the weight of CAC decreased by 8.2% compared to its initial weight. The rate of weight loss of CAC/Ca was faster than that of CAC, with a weight loss of 27.1% at 200 °C. The transition states of CaCl_2_ in CAC/Ca during the desorption process are clearly marked in the Figure 8a. Figure 8b shows the curves obtained by differential scanning calorimetry (DSC) at a temperature increase of 5 °C/min. It can be observed that the DSC curve for CAC showed a single endothermic peak, whereas the curve for CAC/Ca showed two endothermic peaks. The dehydration of the saline solution occurred at 96 °C, while the desorption of the crystalline water occurred at 130 °C. Integration of the DSC curves gave theoretical energy densities for CAC and CAC/Ca of 205 kJ/kg and 783 kJ/kg, respectively.

### 3.2. Heat Output Performance of CAC

#### 3.2.1. Bed Temperature at Different Air Velocities

**Figure 9:**
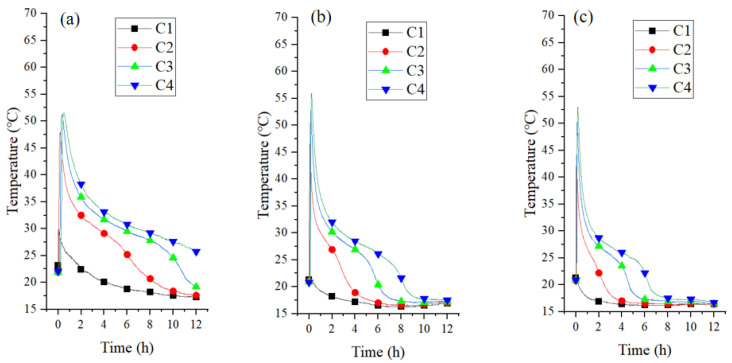
Bed temperature curves of CAC bed at flow rate of 0.5 m/s (**a**), 1.5 m/s (**b**), and 2.5 m/s (**c**).

To gain deeper insight into the heat release behavior, the temperature fluctuations within the reactor were thoroughly examined. Figure 9 illustrates how temperatures evolved over time across various points in the reactor. The reactor schematic (Figure 2b) highlights four representative regions: thermocouples C1 to C4 are positioned sequentially along the bed height to monitor temperature variations at different locations, with C1 nearest the gas inlet and C4 nearest the outlet. Humid air was injected into the reactor at air velocities of 0.5 m^3^/h, 1.5 m^3^/h, and 2.5 m^3^/h. Figure 9a shows that prior to the reaction, the temperatures in the C1, C2, C3, and C4 of the bed were 23.2 °C, 21.9 °C, 21.9 °C, and 22.1 °C, respectively. As the reaction proceeded, the temperatures of four channels increased rapidly to 29.9 °C, 47.9 °C, 51.2 °C, and 51.5 °C in 138 s. Based on sorption mechanisms, elevated sorbent temperatures typically indicate moisture absorption and heat emission processes [45]. Following the peak temperature, a swift drop began, subsequently decelerating to a gradual decline over a period of approximately four hours. Figure 9b,c demonstrate that the temperature trend at different locations was comparable for varying flow rates. The experiment with a flow rate of 1.5 m/s exhibited the greatest temperature increase, reaching 35.1 °C. This phenomenon occurs because increasing the flow rate enhances both the contact frequency of water vapor with the adsorbent and the mass transfer efficiency, leading to faster exothermic adsorption. Specifically, the elevated flow rate accelerates vapor diffusion to adsorption sites, thereby optimizing reaction kinetics.

#### 3.2.2. Temperature and Relative Humidity of Airflow

**Figure 10:**
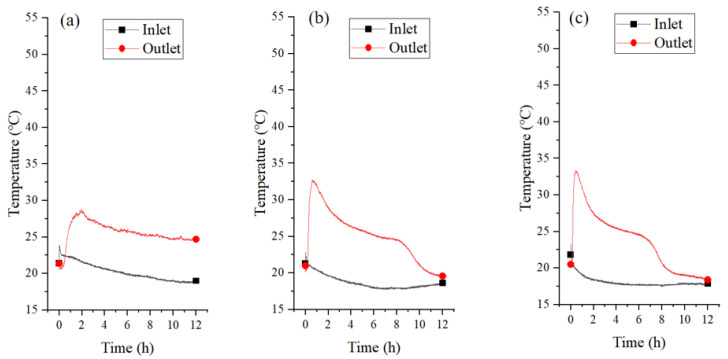
The temperature of inlet and outlet of CAC bed at flow rate of 0.5 m/s (**a**), 1.5 m/s (**b**), and 2.5 m/s (**c**).

Figure 10 and Supplementary Figure S3 illustrate the temperature and RH of the inlet and outlet air flows at different air velocities, respectively. It was observed that the temperature of the inlet flow was 20–24 °C with RH of 70–80%. During the initial reaction phase, the outlet airflow temperature exhibited a rapid increase due to exothermic water vapor adsorption on the CAC surface. This thermal response arose from two coupled mechanisms: (1) localized heat generation at adsorption sites within the porous CAC matrix, and (2) gas–solid convective heat transfer. As adsorption progressed toward saturation, the exothermic heat release diminished, causing a gradual decline in the outlet air temperature. It is found that an exothermic wave within the fixed bed was formed, which was moved from inlet toward the outlet of the reactor [46]. The maximum temperature of the outlet airflow increased from 28.6 °C to 33.2 °C as the air velocity increased from 0.5 m/s to 2.5 m/s. It is evident that an increase in the flow velocity can facilitate the adsorption of water vapor and release of heat, resulting in a higher temperature of the outlet airflow.

#### 3.2.3. Effect of Different Flow Velocities on Discharge Power

**Figure 11:**
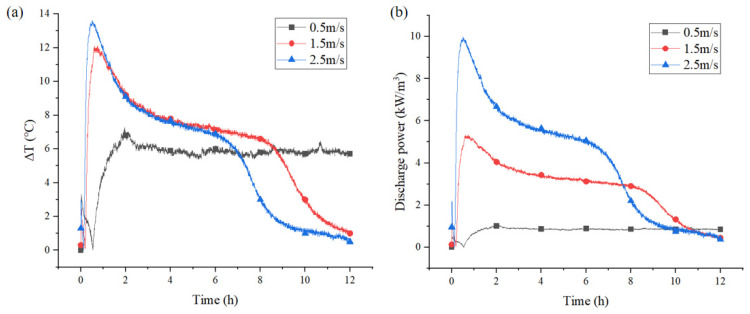
Variations in temperature lift of the air (**a**) and specific discharge power (**b**) with time of CAC bed.

The heat output depended on H_2_O and heat transfer rates, reaction speed, and the heat generated by the reactant mass in the reactor modules [47]. As illustrated in Figure 11a, a higher air velocity was observed to result in an elevated temperature lift between the inlet and outlet airflow. At 0.5 m/s flow velocity, the maximum temperature lift was recorded at 7.3 °C. At 2.5 m/s flow velocity, the maximum temperature lift achieved 13.6 °C. A higher velocity resulted in a more adequate water absorption process for CAC, as well as an enhancement of the heat transfer between the air flow and the solid adsorbent. Furthermore, the exothermic process will be completed in a shorter time. Figure 11b demonstrates the discharge power of CAC in a fixed-bed reactor. At 0.5 m/s air velocity, the peak discharge power was 1 kW/m^3^, increasing to 5 kW/m^3^ at 1.5 m/s. At 2.5 m/s flow velocity, the discharge power increased rapidly to a maximum value of 10 kW/m^3^. Increasing the flow rate provided only marginal enhancement in exothermic power as the result of the low adsorption capacity of CAC. Therefore, loading hydrated salts into CAC was essential to enhance its adsorption capacity and improved its exothermic performance.

### 3.3. Heat Output Performance of CAC/Ca

#### 3.3.1. Bed Temperature at Different Air Velocities

**Figure 12:**
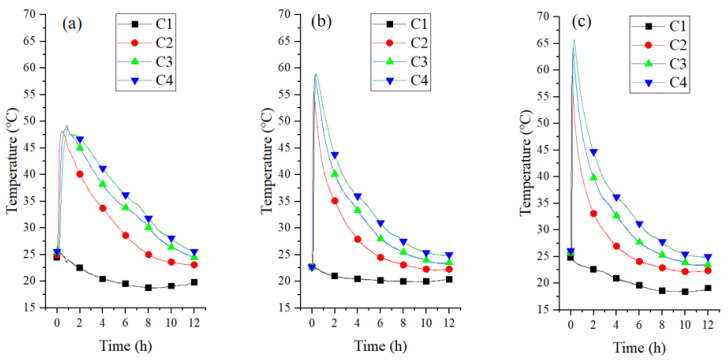
Bed temperature curves of CAC/Ca bed at flow rate of 0.5 m/s (**a**), 1.5 m/s (**b**), and 2.5 m/s (**c**).

Figure 12a depicts the bed temperature curves for CAC/Ca at 0.5 m/s velocity. The temperature of C2, C3, and C4 was rapidly increased from 25 °C to 48 °C, and then gradually decreased to ambient temperature over a 10-h period. Figure 12b,c depict the bed temperature for CAC/Ca under air flow of 1.5 m/s and 2.5 m/s, respectively. At 2.5 m/s air velocity, the maximum bed temperature was 65.7 °C. CAC/Ca exhibited higher temperatures than CAC due to its superior water uptake kinetics and capacity, which directly correlate with the amount of heat released during adsorption. Physical adsorbents and composite adsorbents exhibited distinct adsorption behaviors, each characterized by a unique reaction wave [48]. It was also observed that increasing the air velocity elevates the bed temperature due to accelerated hydration kinetics. Increased air velocity enhances mass transfer—the rate-limiting process governing system thermal performance [49]—by improving water vapor supply to the fixed-bed reactor, thereby increasing hydration conversion.

#### 3.3.2. Temperature and Relative Humidity of Airflow

**Figure 13:**
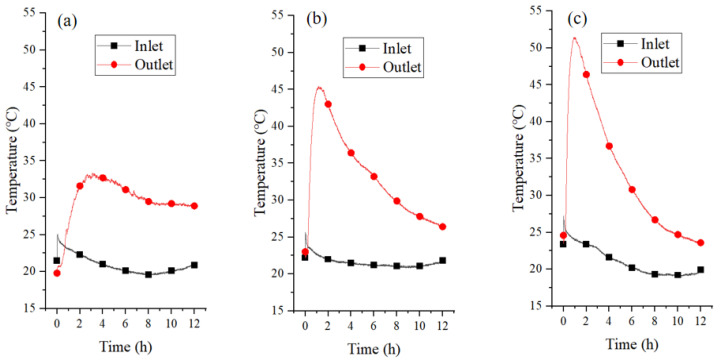
The temperature of inlet and outlet of CAC/Ca bed at flow rate of 0.5 m/s (**a**), 1.5 m/s (**b**), and 2.5 m/s (**c**).

Figure 13 shows the temperature curves of the inlet and outlet airflow for CAC/Ca. As illustrated in Figure 13a, the outlet temperature exhibited a notable increase from 19.8 °C to 33.4 °C within 4 h, subsequently demonstrating a gradual decline to the ambient temperature. Figure 13b,c demonstrated a higher and more rapid outlet temperature in comparison to that of CAC. The maximum outlet temperature at a velocity of 1.5 m/s was 45.4 °C, while the maximum outlet temperature of 51.4 °C was obtained at a velocity of 2.5 m/s. This suggests that a higher flow velocity accelerated hydration reactions and enhanced water uptake. Supplementary Figure S4 shows the RH curves of the inlet and outlet airflow for CAC/Ca. Since calcium chloride occupied the pore structure of CAC, the composite’s adsorption behavior was dominated by the hydration of the salt hydrate, while the CAC matrix enhanced mass transfer and prevented salt leakage. It was also observed that higher inlet air velocities enhanced the exothermic reaction and increased the output temperature by improving both water vapor supply and heat/mass transfer efficiency.

#### 3.3.3. Effect of Different Air Velocities on Discharge Power

**Figure 14:**
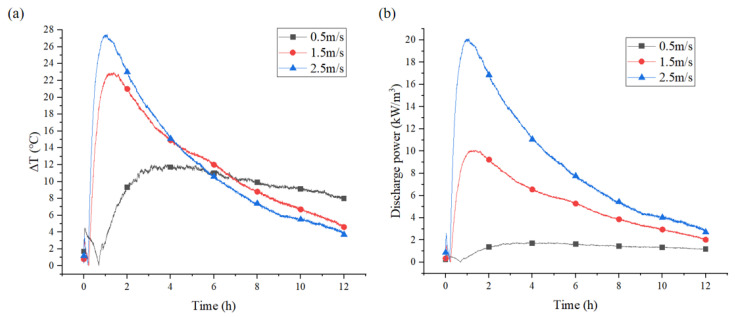
Variations in temperature lift of the air (**a**) and specific discharge power (**b**) with time of CAC/Ca bed.

Figure 14a illustrates the temperature lift of the airflow under different velocity conditions. It was observed that the temperature lift between the inlet and outlet airflow initially increased and then decreased. The maximum temperature lift at an air velocity of 0.5 m/s was 12.0 °C, while the maximum temperature lift at the air velocities of 1.5 and 2.5 m/s was 23 °C and 27.5 °C, respectively. A higher air flow velocity can lead to a substantial rise in the hydration rate [50], so as to obtain a high temperature lift between the inlet and outlet temperature. Moreover, a high air velocity resulted in rapid water vapor supply to CAC/Ca, which possessed a higher water uptake. High flow velocities resulted in efficient heat mass transfer, increasing the temperature lift of the inlet and outlet airflow. Figure 14b demonstrates that the discharge power of CAC/Ca exhibited an upward trend with increasing flow velocity. The highest recorded discharge power was 2 kW/m^3^ at a flow rate of 0.5 m/s. When the flow rate was increased from 1.5 m/s to 2.5 m/s, the maximum exothermic power increased from 10.11 kW/m^3^ to 20 kW/m^3^.

### 3.4. The Comparison of CAC and CAC/Ca

**Figure 15:**
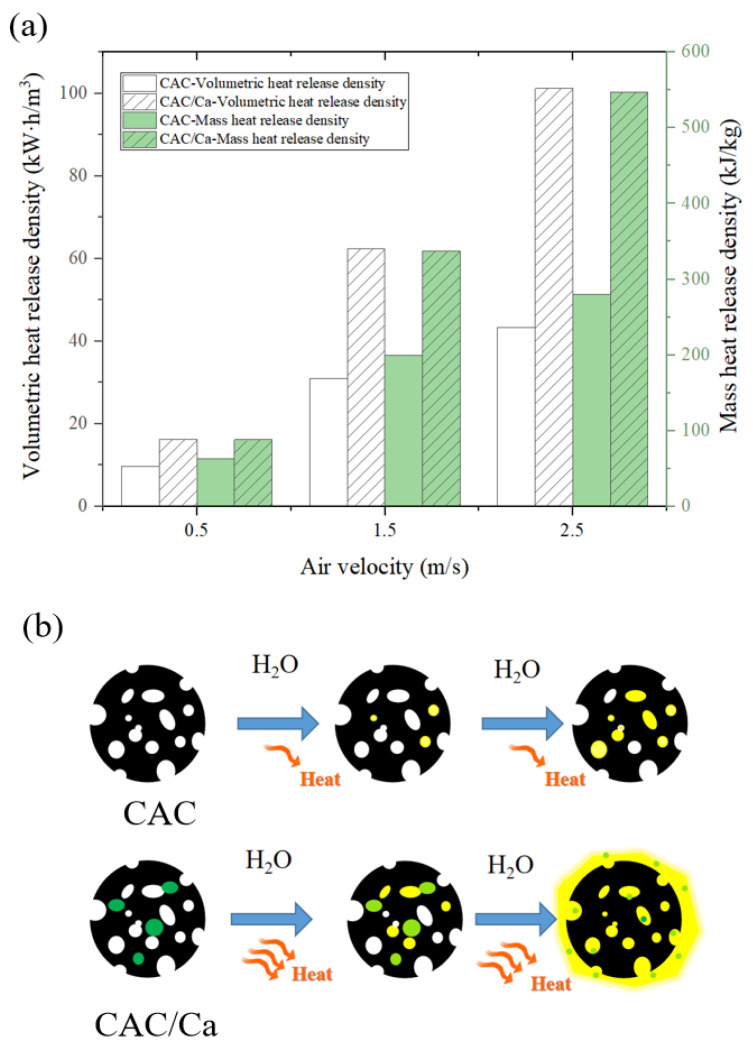
Heat release densities (**a**) and the schematic of the adsorption process (**b**) of CAC and CAC/Ca.

The specific discharge power curves are integrated to obtain the effective heat release amount of the samples in the reactor, as illustrated in Table 2. At flow velocities of 0.5 m/s, 1.5 m/s, and 2.5 m/s, the volumetric heat release density of CAC was 10 kWh/m^3^, 31 kWh/m^3^, and 43 kWh/m^3^, respectively. CAC/Ca demonstrated a significantly higher volumetric heat release density, registering 16 kWh/m^3^, 62 kWh/m^3^, and 101 kWh/m^3^ at flow velocities of 0.5 m/s, 1.5 m/s, and 2.5 m/s, respectively. The mass heat release densities of CAC and CAC/Ca are shown in Figure 15a. The enhanced heat storage density of CAC/Ca stems from chemisorption of CaCl_2_. While the CAC matrix provides physisorption via its mesopores (BET surface area: 231 m^2^/g), the embedded CaCl_2_ enables chemisorption through reversible hydration between CaCl_2_ and H_2_O. The chemisorption allows CAC/Ca to achieve higher water uptake than pure CAC, directly amplifying the thermal energy storage capacity. Furthermore, the CAC matrix mitigates salt agglomeration, ensuring sustained vapor diffusion across cycles, as illustrated in Figure 15b. The heat density of CAC/Ca was found to be 2.15 times higher than that of the CAC at a flow rate of 2.5 m/s. The heat release densities derived from the fixed-bed reactor test and the heat storage densities obtained from the DSC test of CAC and CAC/Ca are presented in Table 3. The heat storage efficiency was determined by calculating the ratio of the heat released during dynamic tests to the heat consumed during DSC analysis. It can be observed that the heat storage efficiencies of CAC and CAC/Ca were 76% and 70%, respectively.

## 4. Conclusions

Ambient humidity-driven adsorption heat storage has shown significant potential for integrated utilization of solar energy and other renewable heat sources. In this study, CAC (representing a physical adsorbent) and CAC/Ca (serving as a composite adsorbent) were systematically investigated to characterize their adsorption thermal release properties. Notably, the composite adsorbent CAC/Ca (containing 24 wt% calcium chloride) exhibited a 4–102% enhancement in water uptake capacity compared to the physical adsorbent, CAC. However, adsorption dynamics were found to be significantly attenuated with increased layer thickness, especially for CAC/Ca. Airflow velocity elevation significantly enhanced thermal output, with CAC/Ca demonstrating superior performance. Increasing velocity from 0.5 to 2.5 m/s induced temperature-lift increments from 7.3 to 13.6 °C for CAC and from 12.0 to 27.5 °C for CAC/Ca. This thermal amplification translated to volumetric discharge powers of 10 kW/m^3^ (CAC) versus 20 kW/m^3^ (CAC/Ca) at 2.5 m/s. Fixed-bed testing further demonstrated that the thermal storage density of CAC/Ca exhibited 547 kJ/kg, which was 3.5-fold higher than that of CAC (156 kJ/kg). This study pioneers a sustainable pathway for biomass-derived thermal energy storage. The developed CAC/Ca composite establishes a new benchmark for low-cost adsorbents that simultaneously addresses energy density limitations and system scalability challenges in intermittent renewable utilization.

## Figures and Tables

**Table 1:** Characterization parameters of CAC and CAC/Ca.

Sample	S_BET_ (m^2^/g)	V_micro_ (cm^3^/g)	V_total_ (m^3^/g)	D_ave_ (nm)	CaCl_2_ Content (%)	Water Uptake 0.2 cm, 5 h (g/g)
CAC	231.26	0.08	0.13	3.8	0	0.09
CAC/Ca	28.74	0.01	0.03	7.0	24	0.17

**Table 2:** Heat release amount (kJ) of CAC and CAC/Ca in the fixed-bed test.

Sample	0.5 m/s	1.5 m/s	2.5 m/s
CAC	18	56	78
CAC/Ca	29	113	183

**Table 3:** Heat storage efficiency of CAC and CAC/Ca.

Sample	Heat Release Density from Fixed-Bed Test/(kJ/kg)	Heat Storage Density from DSC/(kJ/kg)	Heat Storage Efficiency/%
CAC	156	205	76
CAC/Ca	547	783	70

## Data Availability

The original contributions presented in this study are included in the article/Supplementary Materials. Further inquiries can be directed to the corresponding author.

## Supplementary Materials

The following supporting information can be downloaded at: https://www.mdpi.com/article/10.3390/ma18102280/s1, Figure S1. Raman patterns of CAC and CAC/Ca. Figure S2. FTIR spectra of CAC and CAC/Ca. Figure S3. The RH of inlet and outlet of CAC bed at flow rate of 0.5 m/s (a), 1.5m/s (b) and 2.5 m/s (c). Figure S4. The RH of inlet and outlet of CAC/Ca bed at flow rate of 0.5 m/s (a), 1.5m/s (b) and 2.5 m/s (c). Table S1 Comparative table of properties of hydrated salt composite adsorption heat storage materials. References [51,52,53,54,55] are cited in the Supplementary Materials.
