# Supplementary material for: Comparative Assessment of Hygroscopic Properties and Thermal Performance of Activated Carbon-Based Physical Adsorbents and Advanced Composite Adsorbents
Source: Materials (Basel). 2025 May 14;18(10):2280. doi: 10.3390/ma18102280 (PMC12112874; doi:10.3390/ma18102280)
Supplement: Supplementary file 1 [file materials-18-02280-s001.zip › materials-3503917-supplementary.pdf]

Supporting Information

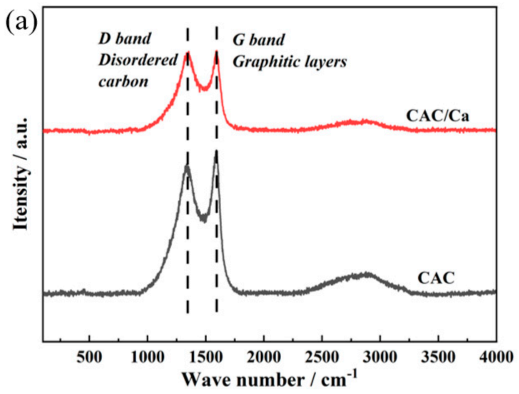

Figure S1. Raman patterns of CAC and CAC/Ca

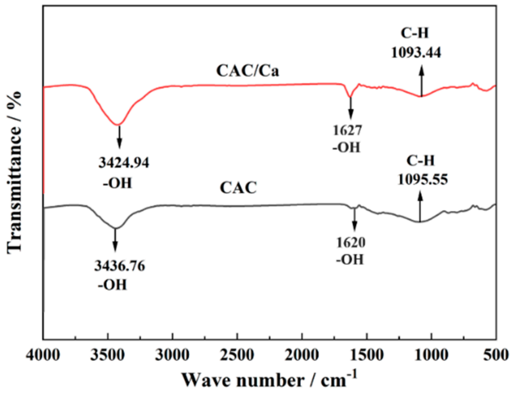

Figure S2. FTIR spectra of CAC and CAC/Ca

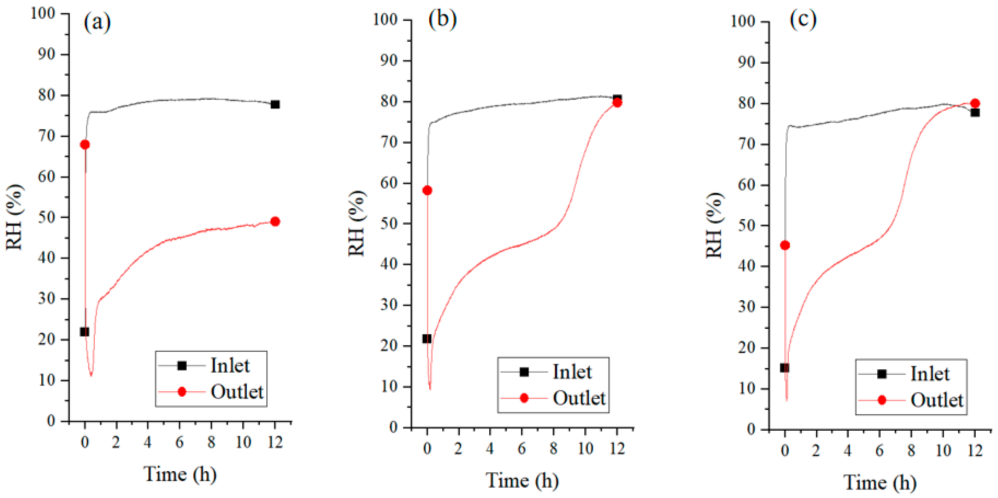

Figure S3. The RH of inlet and outlet of CAC bed at flow rate of 0.5 m/s (a), 1.5m/s (b) and 2.5 m/s (c).

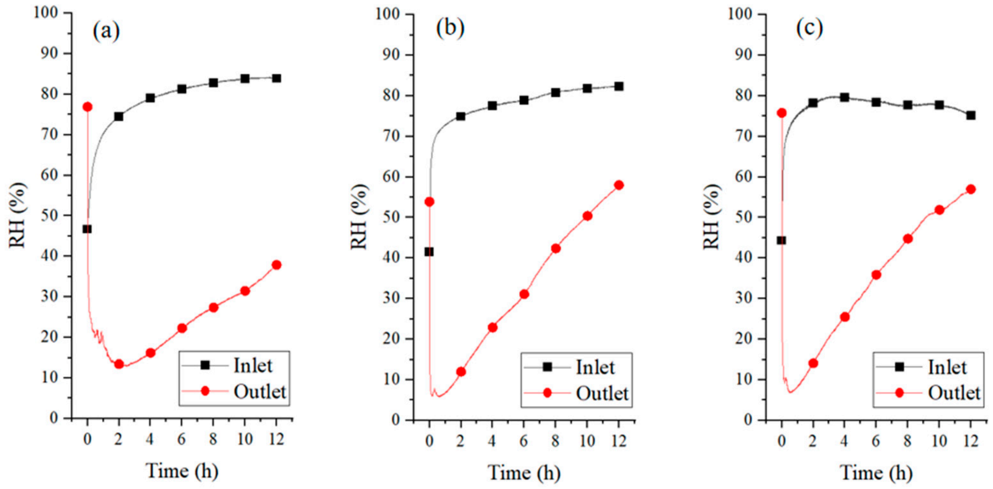

Figure S4. The RH of inlet and outlet of CAC/Ca bed at flow rate of 0.5 m/s (a), 1.5m/s (b) and 2.5 m/s (c).

Table S1 Comparative table of properties of hydrated salt composite adsorption heat storage materials

Composite adsorbents	Matrix	Salt content (wt. %)	Adsorption conditions	Water uptake (g/g)	Energy storage Density (J/g)	Ref.
13X/$MgSO_4$-LiCl	Zeolite 13X	$MgSO_4$: LiCl = 2:1	20°C, 60%RH	0.24	458.3	[51]
VAS	Volcanic	25	25°C, 90%RH	0.79	601.33	[52]
MC/SG/$CaCl_2$	Silica gel	13	50%RH	0.85	---	[53]
EP-30binary	Expanded perlite	30	20°C, 80%RH	1.06	1273	[54]
EP-$CaCl_2$	Expanded perlite	43	53%RH	0.3059 g/ml	0.65 GJ/m^3	[55]
CAC/Ca	Coconut shell activated carbon	24	20 °C, 70% RH	0.37	547	This work

[51] WANG, Z.; XU, X.K.; YAN, T.; Zhang, H.; Wang, L.W.; Pan, W.G. Preparation and thermal properties of zeolite 13X/$MgSO_4$-LiCl binary-salt composite material for sorption heat storage[J]. Applied Thermal Engineering 2024, 245: 122905. doi:10.1016/j.applthermaleng.2024.122905.

[52] WANG, Z.H.; ZHANG, Z.C.; WANG, Y.H.; Li, W.Y.; Liu, S.L. Comprehensive study of a volcanic-based hydrated salt thermochemical energy storage composites for buildings heating in China's low-latitude plateau region: Development, characterisation, and analysis[J]. Journal of Energy Storage 2024, 103: 114396. doi:10.1016/j.est.2024.114396.

[53] SHERVANI, S.; STRONG, C.; TEZEL, F.H. Simultaneous impregnation and microencapsulation of $CaCl_2$ using silica gel and methyl cellulose for thermal energy storage applications[J]. Scientific Reports 2024, 14(1):7183. doi: 10.1038/s41598-023-50672-6.

[54] HU, Z.M.; LUO, J.X.; JIN, Y.H.; Ke, F.L.; Wang, W.; Yin, Q. Experimental study on

thermochemical heat storage performance of expanded perlite-based $SrCl_2/CaCl_2$ binary hydrated salt composites[J]. Journal of Energy Storage 2025, 117: 116199. doi:10.1016/j.est.2025.116199.

[55] CHEN, J.B.; ZHANG, Y.; CHEN, Z.W.; Gan, G.H.; Su, Y.H. Impact of porous host materials on the compromise of thermochemical energy storage performance[J]. Renewable Energy 2025, 245: 122784. doi:10.1016/j.renene.2025.122784.
